# Impact of the COVID-19 pandemic on women’s health nursing clinical practicums in the spring 2020 semester in Korea: a nationwide survey study

**DOI:** 10.4069/kjwhn.2021.09.17.1

**Published:** 2021-09-30

**Authors:** Mijong Kim, Geum Hee Jeong, Hae Sook Park, Sukhee Ahn

**Affiliations:** 1 Department of Nursing, Hannam University, Daejeon, Korea; 2 School of Nursing and Research Institute in Nursing Science, Hallym University, Chuncheon, Korea; 3 Department of Nursing, Dongyang University, Youngju, Korea; 4 College of Nursing, Chungnam National University, Daejeon, Korea

**Keywords:** COVID-19, Clinical competence, Distance education, Nursing education, Republic of Korea

## Abstract

**Purpose:**

This study investigated the impact of coronavirus disease 2019 (COVID-19) on women’s health nursing clinical practicums in undergraduate nursing schools in Korea during the spring 2020 semester.

**Methods:**

A cross-sectional online survey on clinical practicum teaching experiences in the spring 2020 semester was distributed to members of the *Korean Society of Women Health Nursing* (KSWHN) who taught undergraduate nursing. One faculty member from each of 203 institutions was requested to respond and there were no duplicate participants. Seventy-nine participants (38.9%) responded and 74 responses were analyzed. Descriptive statistics were presented for all survey items.

**Results:**

Fifty-two faculty members (70.3%) belonged to universities and 22 (29.7%) taught at colleges. Thirty-eight (51.4%) answered that their institutions had affiliated teaching hospitals. More than half (52.7%) conducted hospital-based clinical practicums either entirely (n=20) or partially (n=19), whereas the rest of them (47.3%) conducted clinical practicums at school or home via online teaching. The typical teaching methods for offline or online education were case conferences, tests or quizzes, scenario studies, nursing skill practicums, (virtual) nursing simulations, and simulated patient education. Most of faculties (93.2%) supported the development of an educational platform to share educational materials and resources, such as case scenarios.

**Conclusion:**

Nursing faculty members utilized various teaching methods to enhance clinical skills and mitigate limited clinical exposure during the early stage of the COVID-19 pandemic. The KSWHN should move forward to develop an education platform and modalities for members who face many challenges related to the accessibility and quality of nursing education contents.

## Introduction

The first coronavirus disease 2019 (COVID-19) patient was reported in Korea in February 2020, triggering chaos in education for the spring semester. Korea’s academic year starts in March, and most elementary, middle schools, and high schools postponed the opening of classes repeatedly, adding to confusion. Universities starting conducting untact (non-face-to-face) online classes in mid-March, without sufficient time for preparation by either learners or instructors [1]. For the accreditation of nursing schools in Korea, nursing students are required to complete at least 1,000 hours of clinical practicum in various fields ranging from in-hospital care to community health. Nursing school faculty were faced with the urgent need to ensure that students could receive clinical practicum experience in the restrictions of a pandemic situation. Some medical schools and nursing schools mandated students’ clinical training in hospitals in the early phase of the COVID-19 pandemic. However, these mandates met protests from students and their parents [2] because of a lack of adequate protection, protective supplies (including masks), and appropriate training [3]. There was also a case of a medical student who was infected with COVID-19 after a week of clinical practicum at a university hospital [4].

The Ministry of Health and Welfare recommended guidelines for students’ clinical practicums: “Clinical schools should determine whether training for students is possible after considering student safety. If necessary, they can postpone training and adjust the academic schedule. If the clinical practicum is impossible, alternative measures such as in-school practice could be provided” [3]. The Ministry of Education also distributed guidelines on safety management for clinical training at hospitals or health centers for preventing, managing, and responding to COVID-19 [1]. At some universities, safety management for students was put in place in accordance with the official guidelines. Therefore, except for a subset of universities that conducted clinical practicums with thorough preparation for the safety of students, most universities operated clinical practicums outside of the hospital (i.e., in school or online).

In the United States, the American Association of Colleges of Nursing (AACN) provided regularly updated guidelines for nursing colleges in response to the spread of COVID-19 [5]. According to the AACN, student nurses are valuable members of the medical team who provide health care in hospitals and community health care settings. The AACN recommends the restriction of direct student care for confirmed or suspected COVID-19 patients to ensure student safety. When clinical practicum is difficult, emergency alternative plans include simulation, telemedicine education service, virtual reality nursing, online materials for educating clinical nursing, and online group chats.

The nursing clinical practicum curriculum for the spring semester of 2020 also had to rely on classroom or non-face-to-face situations in Korea. Nursing educators are thought to have developed and delivered educational content in a way they deemed would optimally enhance the educational effects of nursing clinical practicum education. Therefore, in the absence of concrete guidance, clinical nursing practicums in the spring semester of 2020 might have been very diverse. No research has yet investigated the format, specific content, or exact status of each clinical practicum for nursing students during the first semester of 2020. The details may have also depended on faculty members’ competency and support from the school.

When traditional clinical practicums become difficult in some emergencies, nursing educators may help nursing students obtain face-to-face training with hospital patients. It is necessary to prepare a new clinical practicum method that can replace existing clinical practicum education. This preparation should start with an understanding of the status of nursing clinical practicums during the early COVID-19 pandemic in 2020. The *Korean Society of Women Health Nursing* is the official academic society of nurse educators and researchers in the field of women’s health nursing in Korea and its members include virtually all relevant nursing faculty. Therefore, the authors undertook this survey with support from the Society, to be administered to its members.

This study aimed to identify the status of clinical practicums for women’s health nursing for undergraduate nursing students in the early stage of the COVID-19 pandemic (spring 2020 semester) and to identify the information and resources needed by nursing educators through a survey administered to nursing faculty members teaching women’s health nursing. The specific objectives were as follows: first, to identify the characteristics of the participants, their schools, and women’s health nursing clinical practicums; second, to determine the status of women’s health nursing clinical practicums in the early stage of the COVID-19 pandemic (the spring semester of 2020); third, to identify the most challenging and positive points, and fourth, to elicit information on faculty members’ needs for women’s health nursing practice in the early stages of the COVID-19 pandemic. The findings of this study will provide essential data for seeking countermeasures and alternatives for women’s health nursing clinical practicum education in response to various disaster situations, including infectious disease pandemics.

## Methods

Ethics statement: This study was approved by the Institutional Review Board of Chungnam National University (202008-SB-108-01). Informed consent was obtained from all participants.

### Study design

This cross-sectional survey employed a descriptive observational study design to describe the status of women’s health nursing clinical practicums during the early stage of the COVID-19 pandemic.

### Setting

Data collection for this study was carried out from October to December 2020. The research team sent a group e-mail to members of the Korean Society for Women’s Health Nursing. Google Forms, a free online survey tool, was used. Participants who agreed to participate in the survey accessed the online questionnaire link. As a token of appreciation for participating in the study, one mobile coupon (approximately 5,000 Korean won, equivalent to roughly 4 US dollars) was sent to the respondent if they opted to receive a reward.

### Participants

The target population was all professors who teach women’s health nursing at all universities or colleges nationwide in Korea with an established nursing department (203 as of August 1, 2020). If there was more than one professor of women’s health nursing at the same school, only one person was requested to respond. Participants indicated that they understood the purpose and methods of this study, met the conditions, and voluntarily consented to participate. There was no specific exclusion criterion. E-mails advertising the study and inviting Society members to participate voluntarily were sent via the Society e-mail service up to four times. A total of 79 women’s health nursing professors from 79 nursing schools participated in the survey voluntarily. This corresponds to a response rate of 38.9% out of the 203 schools.

### Data sources/measurement

The research team developed a survey tool through brainstorming to determine the questionnaire items. The team was composed of women’s health nursing professors who had experience educating nursing students during the spring semester of 2020, in the early stage of the COVID-19 pandemic. They finalized the questionnaire items (Supplementary Data 1). No reliability test was done before the survey due to the characteristics of the study.

### Variables

The following items were measured as variables: first, characteristics of participants, educational institutions, and the clinical practicum; second, information on the women’s health nursing clinical practicum in the spring 2020 semester (e.g., credits, number of students per unit, number of professors [instructors]) and credit distribution; third, if there was a hospital practicum, the type of clinical practicum; fourth; information on in-school practicum or non-face-to-face training operations; fifth, opinion on building a content platform for women’s health nursing clinical practicum and intention to share data; and sixth, information on the affiliated educational school and information on full-time professors of women’s health nursing. Finally, the following were asked as open-ended questions: the most difficult points and solutions in clinical practicum education, the most positive points in the clinical practicum during the early stage of the COVID-19 pandemic, and expectations of the Society in relation to the women’s health nursing clinical practicum in the pandemic situation.

### Bias

There was no sampling bias.

### Study size

No sample size estimation was done. The respondents included the entire target population.

### Statistical methods

The collected data were analyzed using IBM SPSS ver. 24.0 for Windows (IBM Corp., Armonk, NY, USA). The characteristics of the participants and the schools with which they were affiliated were analyzed with descriptive statistics such as error, percentage, mean, and standard deviation. The characteristics related to the operation of the women’s health nursing clinical practicum were analyzed using frequency and percentage. The most difficult points, solutions, the most positive points in clinical practicums during the spring 2020 semester, and expectations of the Society were categorized. Opinions on establishing an educational platform for women’s health nursing practice and sharing their data were analyzed using frequency and percentage.

## Results

### Descriptive characteristics of participants and their institutions

Participants’ age and length of educational experience are presented in Table 1, as well as their institutions’ characteristics: regional location, type of nursing school (university or college, number of faculty members of women’s health nursing, presence of affiliated hospitals, clinical practicum credit and hours for women’s health nursing, and the number of students in the clinical practicum course for women’s health nursing) (Table 1). Out of the 74 institutions, 36 (48.7%) had no affiliated hospitals. At 40 institutions (54.1%), women’s health nursing had 3 credits and 3 hours.

### Status of nursing clinical practicums in hospitals

Thiry-nine respondents (52.7%) replied that they conducted clinical practicums in hospitals. Of the 39 schools, 20 schools continued student training in hospitals. As the COVID-19 pandemic worsened, the hospitals began restricting practice wards and limiting the duration, time, and number of students. Therefore, in-school practice was accompanied by hospital training. In contrast, 19 schools switched to in-school training or non-face-to-face training. During the early stage of the COVID-19 pandemic, the most common educational tools were bedside clinical practicums (39), followed by case presentation at meetings, core skills training, exams or quizzes, presentation of educational materials at conferences, and simulation practice training (Table 2).

### Status of in-school or non-face-to-face nursing clinical practicums

Thirty-five schools (47.3%) conducted only in-school or non-face-to-face training for clinical practicums. The other 34 schools also adopted those tools. The most frequently used practice method in in-school practice or non-face-to-face practice was case presentation at a meeting (n=59, 14.5%), followed by tests or quizzes (n=52, 12.8%), self-developed virtual case learning (n=49, 12.0%), core skills training in the lab (n=46, 11.3%), presentation of educational materials (n=41, 10.0%), online core skills training (n=37, 9.1%), online virtual simulation training (n=33, 8.1%), simulation practice (n=29, 7.1%), self-learning simulation practice (n=28, 6.9%), journal club (n=16, 3.9%), special lectures or videos by field nurses (n=11, 2.7%), and the use of electronic medical records (n=6, 1.6%) (Table 3).

Core skills education was conducted using video materials on core skills in women’s health nursing developed by the Korean Society of Women’s Health Nursing. Tests and quizzes were also classified by various respondents. Drug use, term/abbreviation quizzes, and virtual cases in the ward were presented. Online tests were conducted using Google Classroom, Zoom (Zoom Video Communications, San Hose, CA, USA), and the school learning management system. Special lectures by experts in the field of women’s health nursing or field leaders’ ward orientation and nursing situation were video-recorded, and clinical practicums (n=11) were performed to compensate for the limitations of hospital practice.

### Difficulties, positive points, and coping methods for the clinical practicums

#### Difficulties

The respondents reported difficulties in using teaching methods, challenges in developing practical content for in-school and online education and configuring programs due to the changed methods of practical education, challenges in communicating with students during online practicums, and an increase in instruction time relative to the number of practice credits (Table 4).

#### Positive points

As positive results, it was possible to improve the quality of practical education, improve the awareness of infection control, and secure student safety through the application of various teaching and learning methods such as reading articles, developing educational materials, and conducting group discussions or role-plays in various situations (Table 4).

#### Coping methods

Based on experiences in the spring semester, methods of coping in the fall semester included extending the practice time while operating the clinical practicum as much as possible, developing online learning content or purchasing and running a virtual simulation program, and providing various types of educational materials and programs for in-school education. Participants also mentioned development and operation through direct participation of students, and self-learning by students through self-practice time, self-directed learning, and team projects (Table 4).

### Responses on needs for and willingness to share data on an educational platform for women’s health nursing clinical practicums

The items related to building a platform for women’s health nursing practicums are presented in Table 4. The most frequently desired materials were case scenarios (69), case-related information (order sheets, nursing records, and lab sheets) (69), and video clips for cases (68) (Table 5).

Out of 74 participants, 50 (67.6%) expressed willingness to share clinical cases, and 41 (55.4%) indicated that it would be desirable to share the relevant forms required for a case scenario and other materials (Table 5).

## Discussion

### Key results

Thirty-nine (52.7%) nursing schools fully or partially implemented women’s health nursing practice training at the hospital site. Nursing students are members of the medical team in the COVID-19 pandemic; therefore, it is necessary to secure their safety and ensure that they can continue to carry out their roles as members of the treatment team. However, 35 (47.3%) nursing schools did not have practical education in the hospital. The most common reason for this was that due to the COVID-19 pandemic, institutions suddenly requested the suspension of nursing students’ practice. In some other cases, it was difficult to secure space for a course in hospitals. Many in-school or non-face-to-face educational tools were adopted for the clinical practicum to overcome these circumstances. Participants expressed the desire for the Society to provide an adequate educational platform for women’s health nursing.

### Interpretation

#### The paucity of clinical practicum in hospitals

At the beginning of the spring 2020 semester, clinical practicums in the hospitals depended on the presence of a hospital that was affiliated with universities or colleges. Out of 39 institutions where clinical practicums were held, 38 had affiliated hospitals. As the COVID-19 pandemic has continued, clinical practicums in hospitals have been dependent on affiliated hospitals. Under these circumstances, it is difficult to ensure training of students without an affiliated hospital. A nursing college in Singapore formed a network with the government and representatives of medical schools to solve this problem. They established a strong communication and cooperation system to resume practice in hospitals as an essential component of nurturing future nurses [6]. A scarcity of in-hospital practice can lead to a decline in the quality of nursing education. Therefore, it is urgent to establish a solid and close network among nursing education institutions, the government, and medical schools to secure a minimum level of student training in Korea.

#### Non-face-to-face practice methods

During the early stage of the COVID-19 pandemic, 47.3% of nursing universities or colleges replaced clinical practicums with in-school practice or online non-face-to-face practice. If a variety of standardized clinical cases of women’s health nursing were to be developed, educational resources would be used together. For example, there were many responses that the DVD maternity edition [7] video material developed by the Korean Society of Women Health Nursing in 2017 was useful educational material in the current COVID-19 pandemics. The video materials dealt with core skills. The respondents answered that the use of these videos enhanced students’ ability to perform core skills by providing them with sufficient time for self-practice as part of in-school or online non-face-to-face practice (Table 4).

#### Content of practice in clinical skill labs or non-face-to-face practice

The content of in-school or online non-face-to-face practice, which may replace clinical practicums, was as follows: wearing maternity clothes and writing a testimonial, experiencing the delivery process using a delivery simulator, and practicing with a breast massage model and a newborn baby care model The professors responded that through these various learning modules, students’ understanding of nursing recipients and core skill competencies improved. Previous study [8] presented adult nursing cases in online non-face-to-face adult nursing practice, using vSIM® (Laerdal Medical, New York, NY, USA) or online core technology programs, and teaching-learning methods using Google Classroom or Zoom. This is similar to the finding that students’ learning satisfaction and academic achievement increased by improving voluntary participation, particularly as repeated practice of core nursing skills became possible. Therefore, professors should be constantly interested in new models or learning tools and should know how to apply them when necessary. In online non-face-to-face practical education, field leaders and experts developed new educational content such as online core skills and virtual scenario (vSim®) education, medical records, video educational material development, and unique lecture videos. It was difficult for students to acquire on-the-ground practical skills as an observation-oriented course progressed in a hospital [9]. The need for new changes has continued to be raised due to the protection of personal information and difficulties experienced by male students in practicing [10].

The introduction of practical content could compensate for the lack of clinical practicum education conducted in hospitals. Students feel ambivalence toward non-face-to-face practice. A prior study investigated students’ experiences of different situations using online nursing education content through non-face-to-face training. They found that nursing performance improved by integrating previously learned theories into practice, and self-efficacy and confidence improved after core skill training [8]. Nonetheless, there are limitations in observing and practicing the proficiency of medical personnel through online practice, and it is unfortunate that it is not possible to conduct practical training in special departments such as delivery rooms. Therefore, professors need to review and develop suitable practical training methods and educational content to help students obtain the educational effects of in-hospital clinical practicums.

#### Expansion of non-face-to-face education and countermeasures

The COVID-19 pandemic has been a significant crisis in the world of education, as changes to which many curriculum development experts have failed to respond over the past 40 years took place at once. The importance of non-face-to-face education is expected to grow as it becomes a daily routine [6,8]. At nursing colleges in the United States, professors switched to non-face-to-face education in a situation where it was challenging to practice face-to-face education with patients in hospitals, and they conceptualized and provided alternative clinical experiences [11]. Dewart et al. [12] reported that, through online learning, students could gain insight into the impact of infectious diseases on humanity and learn the role of a licensed nurse without providing clinical practicum education to students. Non-face-to-face education can be expanded while supplementing, rather than completely replacing, existing face-to-face education. Social sympathy for non-face-to-face education, sufficient experience and preparation, and supplementation to address problems caused by non-face-to-face education are necessary [13,14].

#### Professor’s burden

In the recent COVID-19 pandemic, the responsibility of professors has increased due to dramatically growing demands for professors to invest time in organizing in-school practice programs to replace in-hospital training and apply various new teaching methods. Forty universities or colleges (54.1%) maintained 3 hours of clinical practicum for 1 credit, and 28 universities or colleges (37.8%) hired two or more full-time women’s health nursing professors. The respondents pointed out the lack of human resources and administrative infrastructure as problems. It will be helpful for professors to share information about other universities’ in-school practice and online non-face-to-face practice cases and personally developed content for women’s health nursing practice to overcome these difficulties.

### Limitations

Despite several attempts to invite Society members to participate in the study, the response rate was 38.9% (79 out of 203 schools), and 74 responses were analyzed. Although participation was relatively even across geographical areas and was not heavily concentrated in a particular region, the results are limited to the nursing schools of the respondents.

In conclusion, this survey of nursing institutions’ experiences of women’s health nursing clinical practicums during the early COVID-19 period found that 69 participants (93.2%) supported building a shared platform for case scenarios, case-related forms, and educational videos. Fifty (67.6%) expressed their intention to share case scenarios, and 41 (55.4%) indicated interest in sharing case-related forms. The *Korean Society for Women’s Health Nursing* or other nursing college educational associations should consider this need and pursue building and operating such a platform to share practical educational content. Such initiatives could also contribute to nursing clinical practicum education by conducting joint research on teaching methods and sharing the results. In addition to challenging traditional educational methods of in-class learning and clinical practicum, the COVID-19 pandemic has also required us to think about continuing education for future nurses. Many changes are expected in health care in the future. The most significant change is the digitalization and smartization of Korea, which will impact the health care industry, leading to a transition to highly specialized medical services. The COVID-19 pandemic is an ongoing crisis that will continue to change the educational environment. Therefore, in the post-coronavirus or with-coronavirus era, universities and professors should be able to establish an educational infrastructure that can perform face-to-face and untact online education in nursing education.

## Figures and Tables

**Table 1.** Characteristics of participants and their institutions (N=74)

Variable	Categories	n (%)
Age (years)	30s	3 (4.0)
	40s	25 (33.8)
	50s	33 (44.6)
	60s	13 (17.6)
Years of education experience	<3	5 (6.8)
	3–4.99	4 (5.4)
	5–9.99	25 (33.8)
	≥10	40 (54.0)
School location	Seoul, Gyeonggi	20 (27.0)
	Daejeon, Chungbuk, Chungnam	13 (17.6)
	Gangwon, Jeju	7 (9.5)
	Daeju, Gyeongbuk	9 (12.1)
	Busan, Ulsan, Gyeongnam	11 (14.9)
	Gwangju, Jeonnam, Jeonbuk	14 (18.9)
School’s nursing education level	BSN level at university	52 (70.3)
	BSN level at college	22 (29.7)
Number of faculty in women’s health nursing	1	46 (62.2)
	≥2	28 (37.8)
Hospital affiliated with university	Yes	38 (51.3)
	No	36 (48.7)
Clinical practicum credit and hours for women’s health nursing	1 credit 1 hour	9 (12.2)
	1 credit 2 hours	15 (20.3)
	1 credit 3 hours	40 (54.0)
	Others	10 (13.5)
Number of students for clinical practicum course for women’s health nursing	<50	10 (13.5)
	50–99	30 (40.5)
	100–149	18 (24.3)
	≥150	16 (21.7)
Number of students per team at clinical placement	≤8	61 (82.4)
	≥9 and more	13 (17.6)

BSN: Bachelor of Science in Nursing.

**Table 2.** Educational tools for in-hospital clinical practicums during the early COVID-19 period in nursing schools in Korea (N=39)

Clinical practicum	n^†^ (%)
Bedside clinical practicum	39 (30.0)
Case presentation	28 (21.5)
Core nursing skills practicum	24 (18.5)
Test (quiz)	18 (13.8)
Development and presentation of material for patient education	14 (10.8)
Nursing simulation	7 (5.4)

COVID-19: Coronavirus disease 2019.

† Multiple responses (n=130).

**Table 3.** Educational methods for in-school or online clinical practicums (N=35)

Educational methods for in-school or online clinical practicums	n^†^ (%)
Case conference	59 (14.5)
Test (quiz)	52 (12.8)
Scenario study (self-developed)	49 (12.0)
Core nursing skills practicum	46 (11.3)
Development and presentation of material for patient education	41 (10.0)
Online core nursing skills practicum	37 (9.1)
Virtual simulation scenario study	33 (8.1)
Nursing simulation practicum	29 (7.1)
Nursing simulation – self-study	28 (6.9)
Journal club	16 (3.9)
Special lecture or prerecorded videos from field nurses	11 (2.7)
Use of electronic medical records	6 (1.6)

† Multiple responses (n=407).

**Table 4.** Difficulties, positive points, and coping methods for clinical practicums by faculty members (N=74)

Variable	Category	n^†^ (%)
Difficulties	Lack of clinical practice time due to the sudden interruption of clinical practicum	19 (25.6)
	Challenges in developing or purchasing practical content for in-school and online education and configuring programs due to the changed methods of practical education	18 (24.3)
	Increase in instruction time compared to the number of practice credits	12 (16.2)
	Challenges in keeping matched clinical sites for women’s health nursing	10 (13.5)
	Using online teaching methods or operating in-school education	8 (10.8)
	Worry about spreading COVID-19 among students	7 (9.4)
	Challenges in communicating with students during online practice operation	4 (5.4)
	Lack of guideline or system how to run clinical education such an emergency situation	3 (4.0)
Positive points	Improving the quality of practical education	13 (17.6)
	Increasing simulation nursing practice or situated scenario-based learning	12 (16.2)
	Improving students’ performance in terms of knowledge, attitude, skills, and nursing processes	11 (14.9)
	Improving the awareness of infection control	7 (9.4)
	Securing student safety through the application of various teaching and learning methods and developing educational materials, conducting group discussions or role-plays of various situations	7 (9.4)
	Increasing the number of interactions between students and faculty members	6 (8.1)
Coping methods	Developing online learning content or purchasing and running a virtual simulation program	25 (33.7)
	Extending the practice time while operating the clinical practicum as much as possible	10 (13.5)
	Providing various types of educational materials and programs for in-school or online education	9 (12.2)
	Developing and operating in-school or online learning through direct participation of students, and self-learning by students through self-practice time, self-directed learning, and team projects	9 (12.2)

COVID-19: Coronavirus disease 2019.

† Multiple responses.

**Table 5.** Necessity of an education platform and willingness to share educational materials (N=74)

Categories	Necessity of an educational platform, n (%)	Willingness to share educational materials, n (%)
Case scenarios	69 (93.2)	50 (67.6)
Case-related information: order sheets, nursing records, lab sheets, etc.	69 (93.2)	41 (55.4)
Video clips for cases	68 (91.9)	33 (44.6)
URLs for YouTube contents	65 (87.8)	30 (40.5)
Video clips for nursing skills	62 (83.8)	34 (45.9)
Information for digital applications	60 (81.1)	28 (37.8)
Test or quiz sheets	60 (81.1)	41 (55.4)
Virtual simulation websites	59 (79.7)	27 (36.5)
Nursing process notes, medication information, video clips for clinical site orientation	19 (25.7)	12 (16.2)

## Supplementary materials

Further details on supplementary materials are presented online (available at https://doi.org/10.4069/kjwhn.2021.09.17.1).
