# Supplementary material for: Impact of the COVID-19 pandemic on women’s health nursing clinical practicums in the spring 2020 semester in Korea: a nationwide survey study
Source: Korean J Women Health Nurs. 2021 Sep 30;27(3):256–64. doi: 10.4069/kjwhn.2021.09.17.1 (PMC9328589; doi:10.4069/kjwhn.2021.09.17.1)
Supplement: Supplementary file 1 [file kjwhn-2021-09-17-1suppl.pdf]

Supplementary materials. Survey on COVID-19 impact on women's health nursing clinical practicums in the spring 2020 semester in Korea: Survey questionnaire

1. 소속 간호학과의 학제는 무엇입니까? *
   ○ 간호학사학위 – 대학
   ○ 간호학사학위 – 전문대학
   ○ 전문학사학위 – 전문대학

2. 소속 간호학과의 1학기 재학생 수(정원외 포함)는 몇명입니까?

3. 2학년 재학생 수

____

4. 3학년 재학생 수

____

5. 4학년 재학생 수

____

1학기 여성건강(모성)간호학실습 학점/시수, 실습분반, 실습 단위(조)별 학생 수, 담당교수(강사) 수와 학점배분 현황에 대한 질문입니다.

6. 1학기 여성건강(모성)간호학 실습교과목 학점과 시수는?
   해당 사항에 모두 표시하세요.
   ☐ 1학점 1시수
   ☐ 1학점 2시수
   ☐ 1학점 3시수
   ☐ 2학점 2시수
   ☐ 2학점 4시수
   ☐ 2학점 6시수
   ☐ 기타

7. 1학기에 여성건강(모성)간호학 실습교과목을 몇 개 분반으로 운영하였나요?(예–1,2,3~개분반) *

____

8. 1학기에 실습교과목을 분반하여 운영한 경우 총 학점은 얼마였나요?(교과목 학점 수* 분반 수) *

____

9. 1학기에 여성건강간호학 실습을 한 총 학생 수는?(전체 학년에서) *

____

10. 1학기에 여성건강간호학실습에서 실습단위(조)별 학생 수는? 예) 8명, 8~9명 *

____

11. 1학기에 여성건강간호학실습 전임교수 수는? *

____

12. 1학기에 여성건강간호학 실습 학점중에서 전임교수가 담당한 학점은?(예, 총 8학점 중 4학점) *

____

13. 1학기에 여성건강간호학실습 실습 강사 수는? *

____

14. 1학기에 여성건강간호학실습 학점 중에서 실습 강사가 담당한 총 학점은?(예: 총 8학점 중 2학점) *

____

지난 1학기 여성건강(모성)간호학 실습 운영의 형태와 실습 시간에 대한 표에 답변을 적어주십시오.

15. 학년별로 실제로 수행한 여성건강(모성)간호학 실습 형태를 선택하여 주세요 *
해당 사항에 모두 표시하세요.

	1) 병원 실습만	2) 교내 실습만	3) 병원 실습+교내실습	4) 비대면 실습 (온라인)만	5) 비대면 실습 (온라인)+교내실습	기타	해당 없음
2학년	☐	☐	☐	☐	☐	☐	☐
3학년	☐	☐	☐	☐	☐	☐	☐
4학년	☐	☐	☐	☐	☐	☐	☐

16. 교내실습의 경우 1학점을 기준으로 할 때, 몇 시수를 운영하셨나요? *
○ 2시간
○ 3시간
○ 기타

이번 학기에 병원 실습을 하셨다면 사용한 실습 형태를 먼저 선택하시고, 그 방법이 차지한 시간 비율을 백분율(%)로 나누어서 표기하고, 지난 해 실습과 비교시 변화된 점을 적어주세요.

17. Q. 병원실습을 하셨습니까?
○ 예 -> 아래 질문에 답해 주세요. (설문지 5)
○ 아니오-> 섹션 7 설문지 6(교내실습)으로 이동합니다
27번째 질문으로 건너뛰세요.

https://doi.org/10.4069/kjwhn.2021.09.17.1

병원실습에 대한 질문. 병원 실습을 하루라도 한 경우 답변해 주세요.

18. 병원실습을 한 경우, 실습 운영형태를 선택하여 주세요 *

	1) 병원실습만	2) 집담회 -사례발표	3) 시뮬레이션 실습교육	4)핵심 술기 실습	5) 집담회 -교육자료 발표	6) 시험- 퀴즈	7) 기타	해당 없음
병원실습	☐	☐	☐	☐	☐	☐	☐	☐

19. 1) 병원 실습: 지난 해 실습 대비 변화된 점

20. 2) 집담회-사례발표: 지난 해 실습 대비 변화된 점

21. 3) 시뮬레이션 실습 교육: 지난 해 실습 대비 변화된 점

22. 4) 핵심술기 실습 : 지난 해 실습 대비 변화된 점

23. 5) 집담회-교육자료 발표: 지난 해 실습 대비 변화된 점

24. 6) 시험(퀴즈) – 지난 해 실습 대비 변화된 점

25. 7) 기타 – 어떤 형태의 실습을 하셨나요?

26. 8) 기타 – 지난 해 실습 대비 변화된 점

27. Q. 교내실습중 실습실 실습을 하셨습니까?
  ○ 예 -> 아래 질문에 답해 주세요
  ○ 아니오-> 아래 질문에 답해 주세요

28. Q. 비대면 온라인실습을 하셨습니까? *
  ○ 예 -> 아래 질문에 답해 주세요.
  ○ 아니오-> 아래 질문에 답해주세요.
  교내실습도 비대면 온라인 실습도 하지 않았다. 42번째 질문으로 건너뛰세요.

교내실습실실습 또는 온라인실습에 대한 질문
교내실습이나 온라인실습을 한번이라도 한 경우 다음 질문에 답변해 주세요

29. 교내실습실 실습 또는 온라인 실습을 한 경우, 실습 운영형태를 선택하여 주세요 *

	교내 실습실 실습	온라인실습	안 함
1) 온라인(리핀코트/엘스비어) 핵심술기 교육	☐	☐	☐
2) 실습실 핵심술기교육	☐	☐	☐
3) 시뮬레이션 실습교육	☐	☐	☐
4) 시뮬레이션 자율실습	☐	☐	☐
5) 가상사례중심 학습(자체개발)	☐	☐	☐
6) 논문 발표	☐	☐	☐
7) 교육자료 발표	☐	☐	☐
8) 가상 시나리오 (V-Sim) 교육	☐	☐	☐
9) 교육용 EMR 이용한 교육	☐	☐	☐
10) 시험 (퀴즈)	☐	☐	☐
11) 집담회 - 사례 발표	☐	☐	☐
12) 기타	☐	☐	☐

30. 1) 온라인 핵심술기(리핀코트/엘스비어) 교육 – 지난 해 실습 대비 변화된 점

____

____

31. 2) 실습실 핵심술기 교육 – 지난 해 실습 대비 변화된 점

____

____

https://doi.org/10.4069/kjwhn.2021.09.17.1

32. 3) 시뮬레이션 실습 교육 – 지난 해 실습 대비 변화된 점

33. 4) 시뮬레이션 자율실습 – 지난 해 실습 대비 변화된 점

34. 5) 가상사례중심 학습(자체개발 시나리오) – 지난 해 실습 대비 변화된 점

35. 6) 논문 발표 – 지난 해 실습 대비 변화된 점

36. 7) 교육자료 발표 – 지난 해 실습 대비 변화된 점

37. 8) 가상 시나리오 (V-SIM) 이용한 교육 – 지난 해 실습 대비 변화된 점

38. 9) 교육용 EMR 이용한 교육– 지난 해 실습 대비 변화된 점

39. 10) 시험(퀴즈)– 지난 해 실습 대비 변화된 점

40. 11) 집담회 – 사례 발표 – 지난 해 실습 대비 변화된 점

41. 12) 기타 운영 형태는 무엇인가요? 지난해 실습 대비 변화된 점은 무엇인가요?
위 사항 외 다른 기타 사항이 있는 경우 자세히 기술해주세요. 지난 해 실습 대비 변화된 점은 무엇인가요?

실습 어려운점과 해결 방안/ 긍정적인 측면과 활용 방안
여성건강간호학 교육자로서 이번 1학기 여성건강(모성)간호학 실습교육에서 가장 어려웠던 점은 무엇이었습니까?
반대로 긍정적인 결과가 있었습니까? 어려웠던 점이나 긍정적인 점을 바탕으로 2학기 실습에서는 어떻게 대처하실 계획입니까?

42. 1. 어려웠던 점 *

43. 1-1. 위의 '어려웠던 점' 에 대해 2학기 실습에서는 어떻게 대처하실 계획인가요? *

44. 2. 코로나19와 관련해서 1학기 여성건강(모성)간호학 실습교육을 하면서긍정적인
결과는 무엇이었습니까? *

45. 2-1. 위의 긍정적인 결과를 높이고자, 2학기 실습에서는어떻게 대처하실 계획인가요? *

여성건강(모성)간호학 실습교육과 관련하여 콘텐츠를 공동으로 사용할 수 있도록 학회 차원의 실습교육 콘텐츠 플랫폼 구축(안)에 대해 어떻게 생각하세요?

46. 여성건강(모성)간호학실습교육콘텐츠 플랫폼 구축 필요성 *
실습교육자료 플랫폼 항목 플랫폼 구축 필요성

사례(시나리오)	필요함	필요하지 않음	모르겠음
사례(시나리오)에 필요한 관련서식(처방지, 간호기록지, 검사결과지 등)	○	○	○
술기 동영상	○	○	○
사례 동영상	○	○	○
핵심 내용 퀴즈	○	○	○
Virtual simulation 웹 공간	○	○	○
Youtube contents URL	○	○	○
실습용 디지털 앱(Application)	○	○	○
기타	○	○	○

https://doi.org/10.4069/kjwhn.2021.09.17.1

47. 실습교육콘텐츠 플랫폼 구축 필요성– 기타

위 '실습교육콘텐츠 플랫폼 구축에 필요한 항목' 외 다른 내용이나 활용방안에 대해 자세히 기재해주세요.

48. 교육자료 공유 의사 *

교육자료 플랫폼 항목 교육자료 공유 의사

사례(시나리오)	필요함	필요하지 않음	모르겠음
사례(시나리오)에 필요한 관련서식(처방지, 간호기록지, 검사결과지 등)	○	○	○
술기 동영상	○	○	○
사례 동영상	○	○	○
핵심 내용 퀴즈	○	○	○
Virtual simulation 웹 공간	○	○	○
Youtube contents URL	○	○	○
실습용 디지털 앱(Application)	○	○	○
기타	○	○	○

49. 교육자료 공유 의사– 기타

위 '교육자료 플랫폼 항목' 외 다른 항목에 대한 공유 의견이 있다면 자세히 기재해주세요.

실습교육과 관련하여 여성건강간호학회에 바라는 사항 : 학술대회나 워크숍 주제를 제안하여 주세요

50. 여성건강간호학실습교육관련 주제 제안

51. 〈모범사례 공유 요청〉 본 학회에서는 동계 교육워크숍에서 코로나19 상황에서 여성건강간호학실습교육 모범사례를 공유하고자 합니다. 공유를 원하는 모범사례가 있다면, 그 사례를 간략히 적고 email 연락처를 남겨주세요. 추후 연락을 드리겠습니다.

※마지막 세션입니다.
다음은 귀하의 일반적 특성에 대한 질문입니다.

52. 1. 귀 대학이 속한 지역은? *
- ○ 1) 서울, 경기
- ○ 2) 대전, 충북, 충남
- ○ 3) 강원, 제주
- ○ 4) 대구, 경북
- ○ 5) 부산, 울산, 경남
- ○ 6) 광주, 전북, 전남

53. 2. 귀 대학의 여성건강간호학 전임교수 수는? *
- ○ 1명
- ○ 2명
- ○ 3명
- ○ 4명 이상

54. 3. 귀하의 연령대는? *
- ○ 30대
- ○ 40대
- ○ 50대
- ○ 60대

55. 4. 귀하의 여성건강간호학 교육경력 *
- ○ 3년 이내
- ○ 3–5년 이내
- ○ 5–10년 이내
- ○ 10년 이상

56. 5. 대학의 부속병원 등 귀 간호학과 학생이 실습할 수 있는 전용 실습기관이 있습니까? *
- ○ 예
- ○ 아니오

* 귀한 시간을 내어 본 연구에 참여해 주셔서 감사합니다. 귀하의 의견을 잘 반영하여 학회 차원의 실습교육 정책을 수립하도록 하겠습니다 *
감사합니다.

https://doi.org/10.4069/kjwhn.2021.09.17.1
